# Progeria syndrome: A case report

**DOI:** 10.4103/0019-5413.38591

**Published:** 2008

**Authors:** Rajul Rastogi, SM Chander Mohan

**Affiliations:** Yash Diagnostic Center, Yash Hospital and Research Center, Civil Lines, Kanth Road, Moradabad - 244 001, Uttar Pradesh, India; 1 Radiodiagnosis and Interventional Radiology, Army Hospital (R and R), Delhi Cantt. - 110 010, India

**Keywords:** Acrogeria, dwarfism, progeria

## Abstract

Progeria is a rare and peculiar combination of dwarfism and premature aging. The incidence is one in several million births. It occurs sporadically and is probably an autosomal recessive syndrome. Though the clinical presentation is usually typical, conventional radiological and biochemical investigations help in confirming the diagnosis. We present a rare case of progeria with most of the radiological features as a pictorial essay.

## INTRODUCTION

Progeria is a rare combination of dwarfism and premature aging. The classic form is known as Hutchinson-Gilford syndrome. It occurs sporadically with a reported incidence of one in eight million births and male predominance with M:F ratio of 1.5:1 and a strong racial susceptibility for Caucasians who represent 97% of patients.^1^

## CASE REPORT

A 14-year-old girl child presented with progressive history of coarsening of skin, failure to thrive and inability to squat for the past three to four years. The child had also developed global alopecia over the past few years. The perinatal history was uneventful. She was apparently normal till one year of age when the parents started noticing the above features. She had normal intelligence. No family history of similar complaints could be elicited.

General examination revealed the child to be of short stature and malnourished. Eyes appeared prominent with hypoplastic chin. Multiple patches of coarse and thickened skin, especially over the dorsum of the hands and shoulders. The terminal ends of the fingers appeared broad and stubby. Based on the history and clinical findings a provisional diagnosis of progeria was made.

**Figure 1:**
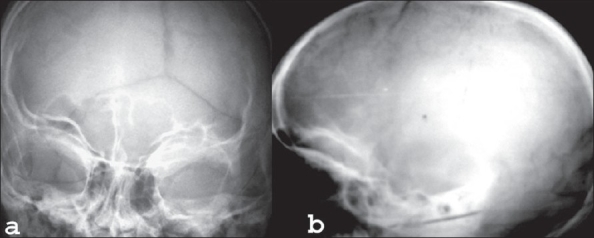
X-ray of the skull, AP (a) and Lateral (b) views showing multiple wormian bones, diastasis of the sagittal suture (a) and prominent vascular markings (b)

**Figure 2:**
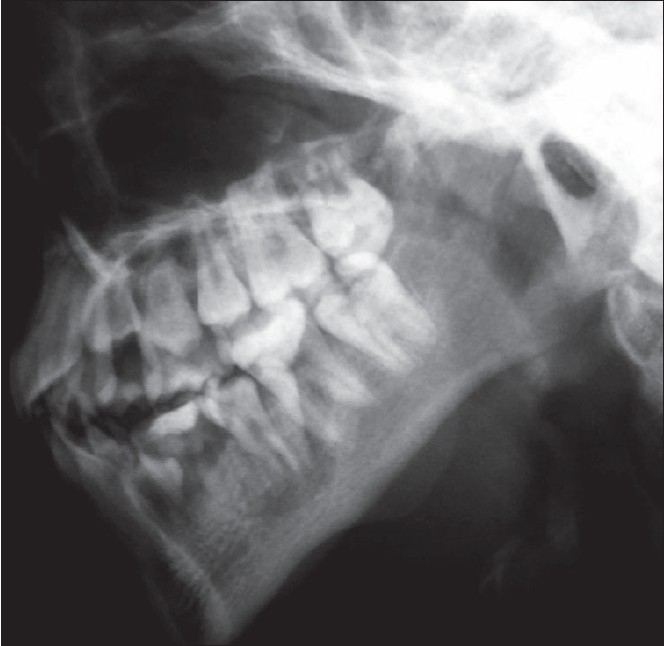
Lateral radiograph of the mandible shows small mandible with small ascending ramus and infantile obtuse angle

**Figure 3:**
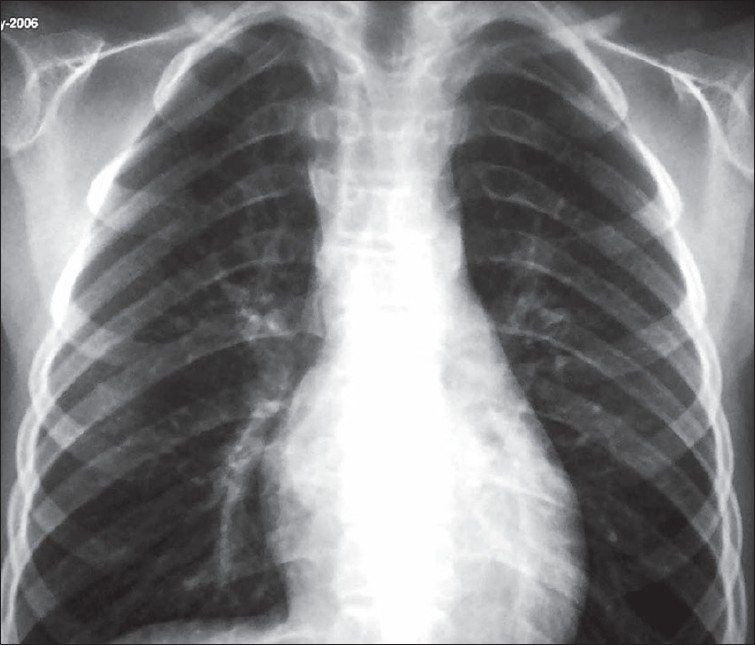
Radiograph of the chest shows absence of the lateral half of the clavicle and thin ribbon-like third rib on both sides

**Figure 4:**
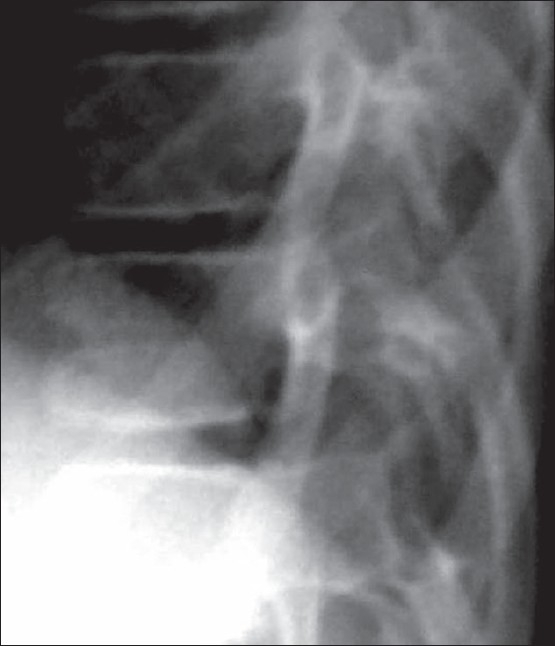
Focused lateral radiograph of the dorsal spine shows fishmouth vertebra

**Figure 5:**
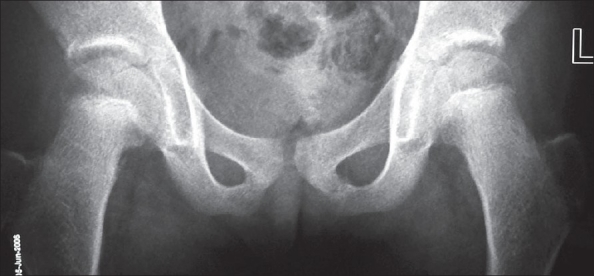
Radiograph of both hips shows severe coxa valga

**Figure 6:**
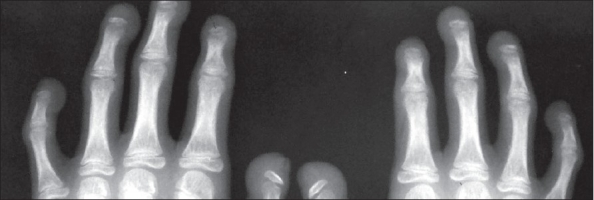
Radiograph of both hands shows acro-osteolysis

Biochemical investigations were normal except for increased serum cholesterol and increased urinary excretion of hyaluronic acid. To confirm the diagnosis, the child was subjected to a skeletal survey. Radiographs of the skull showed diastasis of the sagittal suture with numerous wormian bones [Figure 1]. Radiograph of the mandible showed the hypoplastic mandible with infantile angle [Figure 2]. Radiograph of the chest showed sloping ribbon-like ribs with thinning of both third ribs posteriorly. The lateral half of both the clavicles was absent [Figure 3]. Radiograph of the dorsal spine in the lateral projection showed presence of fish mouth vertebrae [Figure 4]. Pelvis radiograph in AP projection presence of bilateral coxa valga deformity [Figure 5]. Radiograph of the hands and feet revealed resorption of terminal phalanges [Figures 6and7]. The bone age however corresponded to the chronological age of the patient. The radiological findings confirmed the clinical diagnosis of progeria.

**Figure 7:**
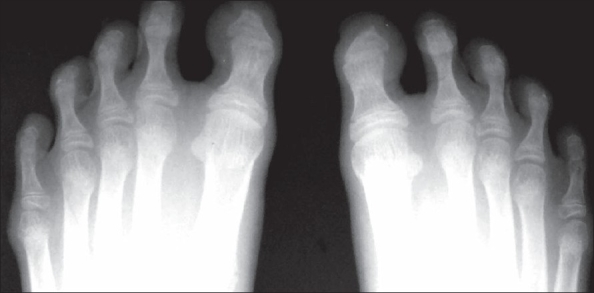
Radiograph of both feet shows acro-osteolysis

## DISCUSSION

Progeria is a genetic disorder rarely encountered and is characterized by features of premature aging.^2^ It is also known as “*Hutchinson-Gilford Progeria syndrome*”. In this syndrome, the rate of ageing is accelerated up to seven times that of normal. The average life span is 13 years (range 7-27 years) occasional survival till the age of 45 years.^3^ The death is mainly due to cardiovascular complications like myocardial infarction or congestive heart failure.^1^

The probable cause is a mutation in the Lamin located in the nuclear matrix.^4^ Increase in the blood hyaluronic acid levels is responsible for sclerodermatous changes and cardiovascular abnormalities. In progeria, rise in blood and serum levels of low-density lipoprotein and cholesterol and total lipids is commonly seen. Failure to thrive may be seen possibly due to a bioinactive growth hormone and lack of vasculogenesis caused by excessive excretion of hyaluronic acid.^5^

The affected children are normal at birth and grow normally till about the end of the first year, when both normal growth and gain in weight slow down. At the end of the first decade, the size attained is that of a normal child of three years of age. Loss of hairs and subcutaneous fat along with sclerodermatous changes give rise to characteristic “*plucked bird*” appearance at about 6-12 months of age. Scalp hair and eyelashes are progressively lost with increased prominence of scalp veins. The affected child is short statured and underweight with an average height of 100 cm and average weight of 12-15 kg or even less.^6^ Progressive degenerative changes occur in the skeleton and blood vessels with advancing age. Delayed eruption and abnormal dentition is also common. The typical “*horse-riding*” stance described in literature is due to the coxa valga deformity. Bone age is normal but mental age may be higher.^1^

Skeletal survey of the patients reveals the following radiological features:^7^ The calvarium is thin and relatively large and the diploic space is absent or very shallow; the face is small with a disproportionate small mandible that retains its infantile obtuse angle. The ascending rami of the mandible are very short. Closure of the anterior fontanelle is delayed. Vascular markings and wormian bones are conspicuous in the large thin calvaria. The clavicles are small in caliber and rarefied at birth; during childhood they may disappear in part or in toto due to progressive osteolysis and fibrosis. The ribs are abnormally gracile and the posterior segments of the upper four ribs on both sides may also disappear in early childhood. The long bones are shortened and overconstricted in their central segments and demonstrate flares at the ends. Coxa valga deformity may be marked, with the neck continuing in the axis of the femoral shafts; the femoral heads are only partially in their acetabular fossae. The greater trochanters are bizarre in shape and position. Some carpal ossification centers are sclerotic, while others participate in the general osteopenia. There may be a marked delay in the healing of fractures and nonunion. Other features include occasional acro-osteolysis and persistence of anterior vascular channels in vertebral bodies.

The differential diagnosis includes Werner syndrome (WS), Acrogeria, Rothmund-Thomson syndrome (RTS) and Cockayne syndrome (CS). Werner syndrome is also known as *progeria adultorum, progeria of the adult and pangeria*. It is the most common of the premature aging disorders. The onset might occur in individuals in their mid-teens or it may be delayed until an individual is as old as 30 years. Both sexes are affected equally. Death usually occurs when patients are aged 30-50 years because of atherosclerosis or malignant tumors. *Acrogeria* is a progeroid syndrome of premature aging of the skin without the involvement of internal organs seen in the Hutchinson-Gilford progeria syndrome. It is seen mainly in females and in the form of sporadic cases. Familial cases are also seen (Gottron type). Acro-osteolysis of the distal phalanges, delayed cranial suture closure with wormian bones, linear lucent defects of the metaphyses and antegonial notching of the mandible are the predominant skeletal features of the disorder.^8^

Rothmund-Thomson syndrome is a hereditary and familial disease characterized by short stature, cataracts, pigmentation of skin, baldness, abnormalities of bones, nails and teeth. Cockayne syndrome spans a spectrum that includes CS Type 1, the classic form; CS Type 2, a more severe form with symptoms present at birth (i.e. cerebrooculofacial-skeletal [COFS] syndrome, Pena-Shokeir Type 2 syndrome); CS Type 3, a milder form; and xeroderma pigmentosa-Cockayne syndrome (XP-CS). Cockayne syndrome Type 1 and Type 2 are autosomal recessive disorders that feature growth deficiency, premature aging and pigmentary retinal degeneration along with a complement of other clinical findings. Type 1 presents at birth, whereas Type 2 appears during early childhood. Fatality usually occurs in early adolescence, but some patients survive until early adulthood.

Till date, no definitive therapy is available and the patient is generally treated conservatively.
